# Protocol paper: a multi-center, double-blinded, randomized, 6-month, placebo-controlled study followed by 12-month open label extension to evaluate the safety and efficacy of Saracatinib in Fibrodysplasia Ossificans Progressiva (STOPFOP)

**DOI:** 10.1186/s12891-022-05471-x

**Published:** 2022-06-01

**Authors:** Bernard J. Smilde, Clemens Stockklausner, Richard Keen, Andrew Whittaker, Alex N. Bullock, Annette von Delft, Natasja M. van Schoor, Paul B. Yu, E. Marelise W. Eekhoff

**Affiliations:** 1 Department of Internal Medicine Section Endocrinology, Amsterdam UMC location Vrije Universiteit Amsterdam, De Boelelaan 1117, 1081HV Amsterdam, The Netherlands; 2 Tissue Function and Regeneration, Amsterdam Movement Sciences, Amsterdam, The Netherlands; 3 Department of Paediatrics, Klinikum Garmisch-Partenkirchen, Garmisch Partenkirchen, Germany; 4 Department of Rheumatology, Royal National Orthopaedic Hospital, London, UK; 5 Emerging Innovations Unit, Discovery Sciences, BioPharmaceuticals R&D, AstraZeneca, Cambridge, UK; 6 Centre for Medicines Discovery, Nuffield Department of Medicine, University of Oxford, Oxford, UK; 7 Oxford Biomedical Research Centre, National Institute for Health Research, University of Oxford, Oxford, UK; 8 Epidemiology and Data Science, Amsterdam UMC location Vrije Universiteit Amsterdam, Amsterdam, The Netherlands; 9 Aging and Later Life, Amsterdam Public Health, Amsterdam, The Netherlands; 10 Cardiovascular Research Center, Massachusetts General Hospital, Boston, USA

**Keywords:** Fibrodysplasia Ossificans Progressiva, Saracatinib, AZD0530, Clinical trial, Drug repurposing, Drug repositioning

## Abstract

**Background:**

Fibrodysplasia Ossificans Progressiva (FOP) is a genetic, progressive and devastating disease characterized by severe heterotopic ossification (HO), loss of mobility and early death. There are no FDA approved medications. The STOPFOP team identified AZD0530 (saracatinib) as a potent inhibitor of the ALK2/*ACVR1*-kinase which is the causative gene for this rare bone disease. AZD0530 was proven to prevent HO formation in FOP mouse models. The STOPFOP trial investigates the repositioning of AZD0530, originally developed for ovarian cancer treatment, to treat patients with FOP.

**Methods:**

The STOPFOP trial is a phase 2a study. It is designed as a European, multicentre, 6-month double blind randomized controlled trial of AZD0530 versus placebo, followed by a 12-month trial comparing open-label extended AZD0530 treatment with natural history data as a control. Enrollment will include 20 FOP patients, aged 18–65 years, with the classic FOP mutation (ALK2 R206H). The primary endpoint is objective change in heterotopic bone volume measured by low-dose whole-body computer tomography (CT) in the RCT phase. Secondary endpoints include ^18^F NaF PET activity and patient reported outcome measures.

**Discussion:**

Clinical trials in rare diseases with limited study populations pose unique challenges. An ideal solution for limiting risks in early clinical studies is drug repositioning – using existing clinical molecules for new disease indications. Using existing assets may also allow a more fluid transition into clinical practice.

With positive study outcome, AZD0530 may provide a therapy for FOP that can be rapidly progressed due to the availability of existing safety data from 28 registered clinical trials with AZD0530 involving over 600 patients.

**Trial registration:**

EudraCT, 2019–003324-20. Registered 16 October 2019, https://www.clinicaltrialsregister.eu/ctr-search/trial/2019-003324-20/NL. Clinicaltrials.gov, NCT04307953. Registered 13 March 2020.

**Supplementary Information:**

The online version contains supplementary material available at 10.1186/s12891-022-05471-x.

## Background

Rare diseases are defined in Europe as disease with a prevalence of less than 1 in 2000. There are more than 7000 rare diseases, cumulatively affecting the lives of an estimated 3.5–5.9% of the global population [1]. Together, rare diseases provide a significant medical, psychological and economic burden, while posing unique and urgent challenges for research and drug development [2, 3].

An especially devastating rare disease is Fibrodysplasia Ossificans Progressiva (FOP): this genetic, disease is characterized by severe heterotopic ossifications (HO) in muscle, tendons and ligaments and has a prevalence of about 1 in 1 million [4, 5]. HO is often, but not always, preceded by inflammatory symptoms (e.g. swelling, pain and redness) which is known as a flare-up [6, 7]. Culminating heterotopic bone results in progressive immobility and morbidity, ultimately leading to a reduction in lifespan to a median of 56 years [8]. FOP is caused by an autosomal dominant germline mutation in the gene *ACVR1* encoding the BMP receptor kinase ALK2), with an overwhelming majority of patients (97%) exhibiting the ‘classical’ gain of function mutation R206H (c.617G > A) [9]. This mutation leads to aberrant signaling in response to activin A, as well as hypersensitivity to BMP ligands culminating in HO [10, 11].

Only an estimated 5% of rare diseases have a form of FDA approved treatment [12]. Unfortunately, FOP is not one of them. The high costs associated with drug development can hinder development of novel therapies, and therefore repositioning of existing drugs becomes an alluring option for treatment of rare diseases. Toxicology reports, pharmaceutical dossiers and clinical safety data have often already been established for these drugs which may significantly reduce development costs and time to market approval [13, 14].

Kinases have emerged as an important therapeutic target in multiple diseases. This has led to the development of a plethora of approved and/or clinically tested small molecule kinase inhibitors [15, 16]. Such a drug repertoire holds promise that some clinical inhibitors might also inhibit ALK2 in FOP, which has been demonstrated for preclinical kinase inhibitors by experiments in FOP mouse models [17, 18]. Williams et al. performed an unbiased screen on clinically tested small-molecule inhibitors which revealed that saracatinib (AZD0530) had low nanomolar binding affinity for ALK2 and could prevent HO in two FOP mouse models [19].

These findings convinced our team that saracatinib (AZD0530) has favourable characteristics as an investigational drug for FOP. If proven to be safe in patients with FOP and effective in preventing the formation of heterotopic ossification, AZD0530 might represent a rapidly translatable therapy for FOP, with the significant advantage of extensive safety data from over 28 registered clinical trials involving over 600 patients available [19]. The Saracatinib trial TO Prevent Fibrodysplasia Ossificans Progressiva (STOPFOP) is an investigator-initiated trial that aims to investigate both the effectiveness and the safety of AZD0530 for use in patients with FOP [20].

## Methods / design

### Study design

This phase 2a study is designed as a European multicentre 6-month double blind randomized controlled trial (RCT) of AZD0530 versus placebo, followed by a 12 month open-label extension phase.

Eligible subjects are randomized 1:1 to one of two groups: Group 1 will undergo treatment with 100 mg AZD0530 once daily for the entire duration of the trial; group 2 will first be given placebo for the first 6 months before starting AZD0530 for 12 months. After undergoing baseline evaluation, the main outcome criteria and safety endpoints are evaluated at 6, 12 and 18 months after first study drug dosing. In addition, participants are evaluated on safety endpoints 3 weeks after first drug dose/switch to open label phase and after 3, 9 and 15 months. There will be three comparison groups: AZD0530 versus placebo in the 6-month RCT phase (A), placebo group (months 1–6) versus the open label extension phase of the same group (months 7–12) (B) and Open label extension group versus age-, gender-, and demographically-matched historic controls (C).

**Fig. 1:**
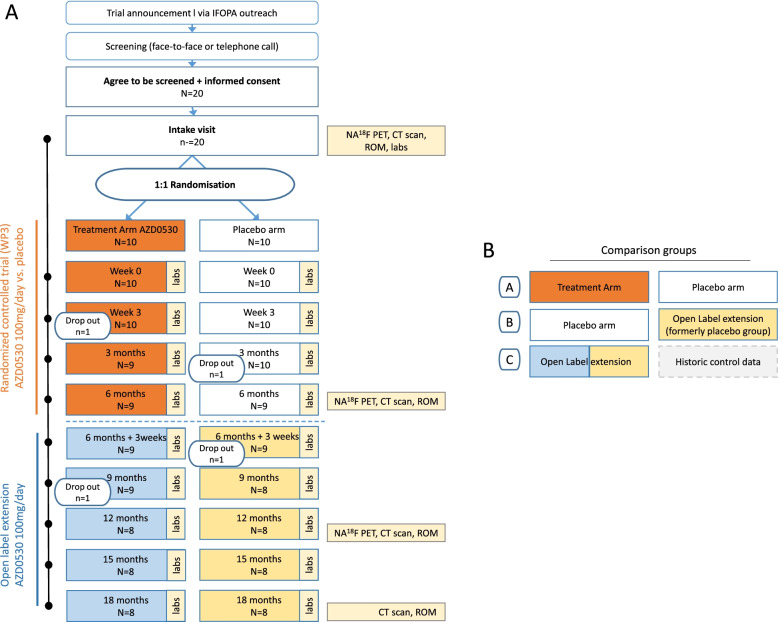
Study overview (**A**) and comparison groups (**B**). IFOPA = International Fibrodysplasia Ossificans. Association, PET = Positrion Emission Tomography, CT = Computer Tomography, ROM = Range Of Motion

Figure 1a summarizes the study design and study procedures, Fig. 1b illustrates the comparison groups.

This study included the first patient in August 2020 and will continue for 18 months after inclusion of the final study participant. Protocol version at time of writing is 12.2. For a completed SPIRIT checklist, see the Additional file 2.

### Participants

Male and female adult (18 years or older) patients with a confirmed diagnosis of FOP caused by the *ACVR1*^R206H/+^ genomic sequence will be eligible for the study. Participants are required to understand and undergo all study procedures. Finally, participants must adhere to effective contraceptive methods throughout the duration of participation in the study.

The exclusion criteria are focused on increasing safety for participants in the study and minimizing risks associated with participation in a clinical study. They include: pregnancy, significant concomitant illness or uncontrolled other illnesses, active bleeding or risk thereof, serious impairment of renal or liver function, and known allergy to any components of the AZD0530 or placebo tablets. Participants cannot simultaneously participate in other interventional trials or studies that use imaging procedures. A full list of patient eligibility criteria can be found in the Additional file 1.

### Recruitment and screening

Recruitment will be performed by dedicated FOP physicians at three of the main FOP centers in Europe: Amsterdam University Medical Centers in The Netherlands, Klinikum Garmisch-Partenkirchen in Germany and Royal National Orthopaedic Hospital in the United Kingdom. Furthermore, patients will be made aware of the trial through national and international FOP patient organisations that are represented in the Stakeholders Board of the study, presentations on patient symposia/webinars and through social media.

At least 1 week prior to the screening visit, patients will receive a full package of study information. After obtaining consent, the patient will be screened for eligibility on site.

#### Randomisation, allocation and (un)blinding

Participants will be randomly assigned to either AZD0530 or placebo group by the clinical trials pharmacy of the Amsterdam UMC. Randomisation will be stratified according to site and performed in blocks of two using a random number table. Patients and study team members will remain blinded until the analysis of study data is completed.

Emergency unblinding can only occur in exceptional circumstances mentioned in Additional file 1.

### Intervention, comparator and concomitant care

Dependent on allocation group, patients will take AZD0530 100 mg once daily or matched placebo orally during 6 months, immediately followed by an open-label extension in which all patients will receive AZD0530 100 mg once daily oral dose for a further 12 months. The placebo is not discernible from the study drug. A placebo comparator was chosen because no FDA/EMA approved drug currently exists for the indication FOP.

#### Discontinuation of study drug

Treatment with study drug will be discontinued by the investigator if a life-threatening AE or a serious adverse event (SAE) occurs; if a pregnancy is confirmed; if the study is closed by the sponsor or regulatory authorities, or if the patient wishes to stop. An elevation of the concentrations of aspartate/alanine transaminase (AST/ALT) of more than five times the upper limit of normal (or three times if there are further signs of severe liver damage) is also a ground for discontinuation. Finally, the drug will be temporarily discontinued if symptomatic SARS-CoV-2 infection occurs, until resolution of symptoms.

#### Adherence

Participants are reminded to take the study drug at the end of the daily diary and at each study visit. Drug adherence will be monitored through pill count at each visit after starting medication. Furthermore, levels of AZD0530 will be monitored at visits 5, 8 and 10.

#### Concomitant care

During the trial, and 2 weeks after the final dose, participants are not allowed to take other drugs that might affect bone turn-over (e.g. bisphosphonates), other experimental drugs, cancer treatments or drugs that cause greater than threefold change in AZD0530 exposure through CYP3A4 interaction.

Escape medication (prednisone and NSAIDs) for exacerbation of FOP is allowed in line with the current FOP guidelines [21].

### Participant timeline

Table 1 shows the schedule of enrollment, intervention, and assessments during the STOPFOP study in accordance with the SPIRIT statement [22]. The flow diagram of the study is shown in Fig. 1a.

**Table 1:**
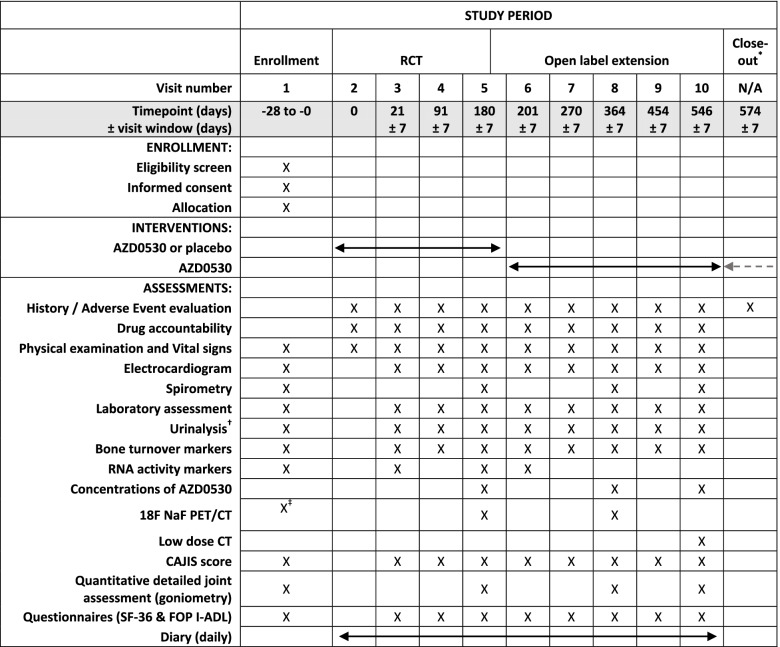
Schedule of enrollment, intervention, and assessments during study

*RCT* Randomized controlled trial, *CAJIS* Cumulative analogue joint involvement scale, *SF-36* 36-item Short Form Survey, *FOP I-ADL* Fibrodysplasia Ossificans Progressiva Instrumental Activities of Daily Living.

*Telephone/virtual visit unless clinically indicated. ^†^Includes pregnancy test for women of childbearing potential. ^‡^No more than 7 days between baseline PET/CT and start study medication

### Endpoints

#### Primary endpoint

The primary endpoint is the change in heterotopic bone volume between baseline and 6-month follow-up as measured by low-dose whole body CT. This difference will be compared between the AZD0530 group and the group that received placebo (comparison group A).

#### Secondary endpoints

Secondary endpoints can be divided into several categories:

- Adverse events: The incidence and severity of adverse events during the different stages of the trial.
- Imaging endpoints including the change in HO volume and FOP activity on ^18^F NaF PET/CT.
- Clinical endpoints such as the number of reported flare-ups through daily diary, cumulative analogue joint involvement scale (CAJIS), Range of Motion, and questionnaires Short Form Health Survey-36 (SF-36) and FOP Independent Activity of Daily Living (FOP-IADL).
- Markers target engagement and efficacy surrogates including bone turn-over biomarkers, AZD0530 pharmacokinetic measurements, and experimental markers.

The outcomes are compared in all three comparison groups.

The endpoints are in line with the agreed Core Outcome Sets by the International Clinical Council on FOP [23]. A detailed description of the endpoints can be found in Additional file 1.

### Data management and data safety monitoring

Data will be handled confidentially. A subject identification code list will be used to link the data to the subject. The handling of personal data will comply with the EU General Data Protection Regulation and any local regulations. Research data will be stored for a period of 20 years.

To review safety and efficacy outcomes during the trial, an independent Data Safety Monitoring Committee (DSMB) will be assembled. The DSMB will consists of independent expert and will include a statistician. The DSMB will primarily monitor safety endpoints, but may recommend also termination of modification of the study in case of interim efficacy of over 75% on the primary endpoint.

Project data will be made publically anonymously available either via the consortium website or via public databases.

### Statistical approach

#### Sample size calculations

Based on the Natural History Study of FOP, progression of HO in FOP is estimated at 120,000 mm^3^ on average per 6-month period with a standard deviation of 48,000 mm^3^ [24]. Expert consensus defines a meaningful decrease in the rate of HO progression as at least 50% reduction in treated vs. control patients, or 60,000 mm3, with a standard deviation of 24,000 mm^3^. Using this projected impact, using a t-test with two-sided alternative, with unequal variances, a trial with treatment and placebo arms of *n* = 8 each would be 80% powered to detect this minimum clinically meaningful difference with an α = 0.05 during the initial 6-month study.

Taking into account an expected drop-out rate of 20%, we calculated the necessary number of participants to be 20 at the start of the study.

#### Analyses of primary endpoint

The primary endpoint of the randomized controlled trial, will be compared by t-test (if normally distributed) or by Mann-Whitney U test (if non-normally distributed) for statistical significance.

We will perform an intention-to-treat analysis including all randomized individuals having data on the outcome (primary or secondary). In addition, we will perform per-protocol analyses including all participants who took at least 80% of dosages.

#### Analyses of secondary endpoints

To analyse the secondary endpoints of radiographic HO progression across all time points in comparison groups B and C, we will use linear mixed models for statistical significance.

Changes in whole body ^18^F NaF SUV will be measured in comparison groups A and B and compared by t-test (if normally distributed) or by Mann-Whitney U test (if non-normally distributed) for statistical significance.

Changes in CAJIS, SF-36 Quality of Life, range of motion surveys and the FOP Independent Activity of Daily Living (FOP I-ADL) will be compared in comparison groups A and B by linear mixed models. The impact of treatment on total joint mobility (measured as positive and negative changes in degrees of range of movement (ROM) for each axis), and changes in the mobility of specific subsets of joints, will be tested in comparison groups A and B via linear mixed models wherever possible.

The correlation between changes in joint ROM and radiographic progression of HO by whole body CT will be tested by Spearman’s rank correlation.

#### Adverse events

Adverse event rates will be coded by body system and MedDRA classification term. Adverse events will be tabulated by treatment group and will include the number of patients for whom the event occurred, the rate of occurrence, and the severity and relationship to study drug.

#### Missing data

In case of many missing data points (defined as over > 20% of cases for primary and secondary analyses), we will consider using multiple imputation techniques as described in the simulation study of Barnes [25].

## Discussion

FOP is a disease that causes heterotopic ossification of muscles, tendons and ligaments culminating in severe morbidity and early mortality in affected patients. Like many rare diseases, there are currently no effective treatments available. The STOPFOP trial aims to investigate the effectiveness and safety of the drug AZD0530 for inhibition of HO in patients with FOP. To our knowledge, this is the first publication on the design of a clinical trial in FOP.

Investigating novel drug therapies in FOP poses several unique challenges that we have tried to mitigate in this trial. Firstly, rare diseases by their very definition have a limited number of patients, often distributed across the globe. The low prevalence of FOP, estimated to be 1 in 1 million [4, 5, 26], and that only a subset of these patients will be both willing to participate and eligible for participation, the STOPFOP project opted for a RCT design combined with open label extension and comparison to a natural history data. This strategy allows for increased statistical power with a lower number of patients. It also allows all participating patients to receive the active study drug, which is a significant motivating factor for participation. Secondly, FOP patients have a particular sensitivity to certain study procedures [23]. Exacerbations of FOP, called flare-ups, can be provoked by a range of traumas and inflammatory events [21]. This includes medical procedures, such as biopsies and intramuscular injections [27]. To that end, our study procedures are aimed to be as non-invasive as possible. Both established imaging methods such as low-dose whole-body CT (without intravenous contrast), as well as novel imaging methods such as the ^18^F NaF PET-CT, allow for non-invasive evaluation of efficacy [6]. All procedures are performed in specialised FOP centers that have medical and paramedical personnel that know how to approach these patients, which minimises risk for patients. Thirdly, FOP is a slowly progressive disease with significant heterogeneity in clinical progression [24]. This poses challenges for using hard clinical endpoints such as mortality or progression-free survival. It also poses a risk for heterogeneity in the rate and extent of response to investigative treatments. To mitigate these issues, the study adheres to objective measurements, of disease including the amount of HO on whole-body CT, biological activity of FOP using ^18^F NaF PET/CT, and the set of core outcomes that have been set by the International Clinical Council on FOP [23]. In addition, the study is restricted to individuals displaying the classic ALK2 R206H mutation, as other less frequent ALK2 mutations associated with non-classical forms of FOP exhibit substantial variability in natural history and severity [9, 28]. Due to the limited number of available study subjects, there is always a risk of underpowering in studies on ultra-rare diseases. Finally, progression of translational research to clinical drug development and hopefully onwards to novel drug therapies for FOP and other (ultra) rare diseases is encumbered by the arduous and expensive nature of drug development combined with a low success rate. Despite legislative efforts to alter the risk-benefit ratio for these drugs, only a small percentage of rare diseases have a form of treatment approved by regulatory authorities [12]. Thus, drug repositioning – using existing clinical molecules for new disease indications - represents an ideal solution for limiting risks in early clinical studies for rare diseases. This has driven the development of this investigator-initiated trial with a unique cooperation of European and transatlantic dedicated FOP researchers, physicians and patient organizations. In the event of a positive study outcome from the STOPFOP study, AZD0530 may represent a therapy for FOP that can be rapidly progressed due to the availability of extensive safety data from 28 registered clinical trials involving over 600 patients.

## Supplementary Information

**Additional file 1.**

**Additional file 2.**

## Data Availability

Data sharing not applicable to this article as no datasets were generated or analysed during the current study.

## Abbreviations

FOP: Fibrodysplasia Ossificans Progressiva
STOPFOP: Saracatinib Trial tO Prevent Fibrodysplasia Ossificans Progressiva
HO: Heterotopic Ossification
ACVR1: Activin A Receptor, type 1
ALK2: Activin receptor-Like Kinase-2
FDA: The United States Food and Drug Administration
EMA: European Medicines Agency
RCT: Randomized Controlled Trial
UMC: University Medical Center
AE: Adverse Event
SAE: Serious Adverse Event
AST/ALT: ASpartate/ALanine Transaminase
SARS-CoV-2: Severe Acute Respiratory Syndrome COronaVirus 2
CYP3A4: Cytochrome P450 3A4
NSAID: Non-Steroidal Anti-Inflammatory Drug
SPIRIT: Standard Protocol Items: Recommendations for Interventional Trials
CT: Computer Tomography
NaF PET: Sodium Fluoride Positive Emission Tomography
CAJIS: Cumulative Analogue Joint Involvement scale
SF-36: Short Form Health Survey-36
FOP-IADL: Fibrodysplasia Ossificans Progressiva Independent Activity of Daily Living
DSMB: Data Safety Monitoring Board
ROM: Range of Motion
MedDRA: Medical Dictionary for Regulatory Activities
