# Supplementary material for: Protocol paper: a multi-center, double-blinded, randomized, 6-month, placebo-controlled study followed by 12-month open label extension to evaluate the safety and efficacy of Saracatinib in Fibrodysplasia Ossificans Progressiva (STOPFOP)
Source: BMC Musculoskelet Disord. 2022 Jun 1;23:519. doi: 10.1186/s12891-022-05471-x (PMC9156821; doi:10.1186/s12891-022-05471-x)
Supplement: Supplementary file 1 — Additional file 1. [file 12891_2022_5471_MOESM1_ESM.docx]

Eligibility criteria STOPFOP

## Inclusion criteria

In order to be eligible to participate in this study, a subject must meet all of the following criteria:

1. Male or female aged 18-65 with a clinical diagnosis of FOP at screening, including congenital malformation of the great toes and a history of spontaneous or injury-induced heterotopic ossification (HO), and have a confirmed classic FOP phenotype by the documentation of an *ACVR1^R206H/+^* genomic sequence.

### Female participants who are women of child-bearing potential will be required to use a highly effective method of contraception as defined in section 5.4, in combination with a condom or diaphragm or cervical/vault caps with spermicidal foam/gel/film/suppository), from the time of enrolment until 4 weeks after final dose of study drug, unless practicing true sexual abstinence as defined in section 5.4.

### Male participants will be required to avoid procreative sexual intercourse with women of child-bearing potential from time of enrollment until 4 weeks after final dose of study drug through use of highly effective contraceptive methods. Male participants with a pregnant female partner will be required to use a condom for the duration of the study and for 4 weeks final dose of study drug. Male study participants will not be permitted to donate sperm for from the time of enrolment and until 4 weeks after final dose of study drug.

1. Participants will have to be able to understand and complete study and willing to sign informed consent (IC). They have to be able to attend and comply with the study visits and related activities, adhere to all study-related restrictions, and able to undergo procedures such as PET and CT imaging.

## Exclusion criteria

A potential subject who meets any of the following criteria will be excluded from participation in this study:

### Not willing to strictly adhere to the reproductive restrictions as defined in section 5.4

### Women who are pregnant or breast-feeding (from the time 3 months prior to 4 weeksafter completion of participation in the study)

### The presence of significant concomitant illness or history of significant illness such as cardiac, respiratory, renal, rheumatologic, neurologic, psychiatric, endocrine, metabolic, lymphatic disease, or infectious disease, that might confound the results of the study or pose additional risk to the patient;

### Evidence of active bleeding (including hematuria or hematochezia,) acute or chronic gastrointestinal illness, inflammatory bowel disease, or mucositis

### Malignant disease / cancer requiring treatment in the past 3 years (except some primary non‑melanoma skin cancer);

### Severely impaired renal function defined as estimated glomerular filtration rate <30 mL/min/1.73 m^2^ calculated by the Modification of Diet in Renal Disease equation;

### Showing uncontrolled diabetes mellitus with an HbA1C > 9%;

### Significant viral illness or active infections at screening or randomisation; Subjects should not have subacute or acute fevers of >101°F (>38.3°C) at time of screening or randomisation

### Evidence of prolonged QT interval at screening or randomization (defined as QTc of >450 ms) .or known congenital long-QT syndrome.

### Neutropenia defined as an absolute neutrophil count of <1,500/µl,

### Thrombocytopenia defined as platelet count <100 × 10^3^/µl,

### Current blood clotting or bleeding disorder, or significantly abnormal INR-prothrombin time or partial thromboplastin time at screening, or clinically significant abnormalities in other screening laboratories, including significant abnormalities in vitamin B12 or thyroid function tests would be cause for exclusion.-

### Abnormal liver function test results defined as aspartate aminotransferase (AST) >2.0 x upper limit of normal (ULN); alanine aminotransferase (ALT) >2.0 x ULN; and / or total bilirubin >1.5 x ULN;

### Known allergy or intolerance to AZD0530 or any excipients used in the investigational medicinal products (see page 104 of the IMPD for the tablet and coating composition)

1. Simultaneous participation in another interventional clinical study or a non-interventional study with imaging measures or invasive procedures (eg. collection of blood or tissue samples); Participation in the FOP Connection Registry or other studies in which patients completed study questionnaires are possible.

### Treatment with another investigational or drug that might interfere with HO formation and the interpretation of the study drug in the last 90 days

### Current use or history of regular alcohol consumption exceeding 14 units/week (6 glasses of 13.0% wine (175ml), 6 pints of 4.0% lager or ale (568ml), 5 pints of 4.5% cider (568 ml) or 14 glasses of 10.0% spirits (25ml)) within 6 months of screening.

1. Currently active metabolic bone disease, other than FOP.

Study endpoints STOPFOP

## Study parameters/endpoints

The following demographic variables at screening will be summarized by dose level: race (for possible race-dependent reaction to study drug) , gender, age, height and weight as clinical and medical history and characteristics.

### Main study parameter/endpoint

The primary endpoint of the study is:

# The objective change between the two arms measured in heterotopic bone volume measured by low-dose whole body CT over the initial 6 month RCT;

### Secondary study parameters/endpoints (if applicable)

- Safety and tolerability assessments are the incidence and severity of adverse events (AE) during the RCT at the end of week 28. It will be an assessment of the net adverse clinical events with regard to safety, efficacy and Qol, but only if the components are well balanced, which will be assessed by the Data Safety Monitoring Board (DSMB) and at the end of the study in collaboration with the STOPFOP.team

- The change in heterotopic bone volume measured by low-dose whole body CT over six-months treatment during open-label extension of AZD0530 compared to the previous placebo arm of the RCT
- The change in heterotopic bone volume measured by low-dose whole body CT over twelve-months treatment during open-label extension of AZD0530 compared to historical data.
- Change in the volume of individual HO lesions measured by low-dose whole body CT over the initial 6 month RCT and in addition the change over twelve-months therapy during open-label extension of AZD0530 compared to historical data and compared to the 6 months placebo-arm.
- Change in number of HO lesions measured by CT over the initial 6 month RCT and in addition the change over twelve-months during open-label extension of AZD0530 compared to historical data and compared to the 6 months placebo-arm.
- In patients with at least 1 active lesion at baseline: Change (and AUC analysis) of lesion activity by ^18^F-NaF PET over the initial 6 month RCT and over months 6-12 compared to the 6 months RCT of the placebo arm, including change from baseline in ^18^F-NaF Standard Uptake Volume (SUV_mean or peak)_ of individual active HO site
- In patients with at least 1 active lesion at baseline:Change in number of active lesion on ^18^F-NaF PET from baseline to 6 and 12months
- In patients with at least 1 active lesion at baseline: Change in number of lesions that are only ^18^F-NaF PET detectable at baseline to CT detectable lesions at 12 months
- Change and percent change from baseline in biomarkers of bone formation levels in serum over time, including Total Procollagen Type 1 N-Terminal Propeptide (P1NP), Alkaline Phosphatase (AP) fasting cross-linked C-terminal telopeptide of type I collagen (βCTX). Selected genetic (DNA- and (mi/m)RNA) markers for FOP activity
- Joint function assessment by physician at baseline and week 3, month 3,6,9,12,and18 by the cumulative analog joint involvement scale (CAJIS) and the quantitative detailed multi-joint assessment at baseline and month 6, 12 and 18.
- Patient-reported global health status the 36-item Short Form Health Survey (SF-36) at baseline and week 3, month 3,6,9,12,and 18
- FOP disease activity assessed by movement disabilities and quality of life using FOP Independent Activity of Daily Living (FOP I-ADL)
- Number of reported flare-ups by the patient (diary by Castor questionnaire)
- Pharmacokinetic measurements: blood for determination of plasma concentrations of AZD0530 (pre-dose)on the day of the study visits at 6, 12

and 18months

Emergency Deblinding

To maintain the overall quality and legitimacy of the clinical trial, code breaks should occur only in exceptional circumstances when knowledge of the actual treatment is absolutely es-sential for further management of the patient. Investigators are encouraged to discuss with the international PI if he/she believes that unblinding is necessary, wherever possible.

If unblinding is deemed to be necessary, the investigator should use the numbers below for emergency unblinding through the Amsterdam UMC clinical trials pharmacy who will provide immediate unblinding information.

Only the affected patient will be unblinded. The Investigator is encouraged to maintain the blind as far as possible. The actual allocation must NOT be disclosed to the patient and/or other study personnel including other site per-sonnel, monitors or project office staff, unless this is absolutely essential for further manage-ment of the patient. The Investigator must report all code breaks (with reason) as they occur on the corresponding CRF page.
